# Newborn white matter microstructure moderates the association between maternal postpartum depressive symptoms and infant negative reactivity

**DOI:** 10.1093/scan/nsaa081

**Published:** 2020-06-24

**Authors:** Saara Nolvi, Jetro J Tuulari, Tuomas Lavonius, Noora M Scheinin, Satu J Lehtola, Maria Lavonius, Harri Merisaari, Jani Saunavaara, Riikka Korja, Eeva-Leena Kataja, Juho Pelto, Riitta Parkkola, Linnea Karlsson, Hasse Karlsson

**Affiliations:** 1 FinnBrain Birth Cohort Study, Turku Brain and Mind Center, Department of Clinical Medicine, University of Turku, Turku, Finland; 2 Department of Medical Psychology, Charité – Universitätsmedizin Berlin, Berlin, Germany; 3 Department of Psychiatry, Turku University Hospital and University of Turku, Turku, Finland; 4 Department of Psychiatry, University of Oxford, Oxford, UK; 5 Department of Future Technologies, University of Turku, Turku, Finland; 6 Department of Biomedical Engineering, Case Western Reserve University, Cleveland, OH, USA; 7 Department of Medical Physics, Turku University Hospital, Turku, Finland; 8 Department of Psychology and Speech-Language Pathology, University of Turku, Turku, Finland; 9 Department of Radiology, Turku University Hospital and University of Turku, Turku, Finland; 10 Department of Child Psychiatry, Turku University Hospital and University of Turku, Turku, Finland; 11 Centre for Population Health Research, University of Turku and Turku University Hospital, Turku, Finland; 12 Turku Institute for Advanced Studies, University of Turku, Turku, Finland

**Keywords:** DTI, maternal depression, infancy, negative affect, susceptibility

## Abstract

Maternal postpartum depression is a prominent risk factor for aberrant child socioemotional development, but there is little understanding about the neural phenotypes that underlie infant sensitivity to maternal depression. We examined whether newborn white matter fractional anisotropy (FA), a measure of white matter maturity, moderates the association between maternal postpartum depressive symptoms and infant negative reactivity at 6 months. Participants were 80 mother–infant dyads participating in a prospective population-based cohort, and included families whose newborns underwent a magnetic resonance/diffusion tensor imaging scan at 2–5 weeks of age and whose mothers reported their own depressive symptoms at 3 and 6 months postpartum and infant negative emotional reactivity at 6 months. The whole-brain FA moderated the association between maternal depressive symptoms and mother-reported infant negative reactivity at 6 months after adjusting for the covariates. Maternal depressive symptoms were positively related to infant negative reactivity among infants with high or average FA in the whole brain and in corpus callosum and cingulum, but not among those with low FA. The link between maternal depressive symptoms and infant negative reactivity was moderated by newborn FA. The variation in white matter microstructure might play a role in child susceptibility to parental distress.

## Introduction

Because the first years of life are periods of dynamic central nervous system (CNS) development (Markant and Thomas, 2013), identifying who is susceptible to early stress exposure is central to the prevention of several psychiatric disorders (O’Donnell and Meaney, 2017). Importantly, stress exposures may cause adverse effects only in some individuals more susceptible to be affected by environmental adversities (i.e. diathesis-stress model) but these individuals may also benefit from good quality environments, as proposed by different theories reflecting environmental sensitivity (Belsky and Pluess, 2009; Boyce, 2016; Greven *et al.,* 2019).

One important early life exposure for infants is the level of maternal postpartum depressive symptoms, the prevalence of which ranges from 10 to 20% in Western societies (Andersson *et al.,* 2006). Maternal depressive symptoms of varying severity reportedly affect maternal caregiving (Field, 2010), child development (Luoma *et al.,* 2001; Liu *et al.,* 2017) and offspring brain development (Meaney, 2018). Negative reactivity, in turn, is a temperament trait linked to higher risk for psychopathology later in life (De Pauw and Mervielde, 2010; Kostyrka-Allchorne *et al.,* 2019). Importantly, maternal depression is related to higher infant negative emotional reactivity (Ramchandani *et al.,* 2011; Kingston *et al.,* 2012). However, studies reporting heightened emotional reactivity after exposure to parental postpartum depression typically also reported modest effect sizes. The interindividual variation in child susceptibility (i.e. children being disproportionally affected by environmental exposures such as parental distress; Meaney, 2018) may explain part of this variability.

The mechanisms of susceptibility likely include genetic and behavioral factors (Belsky and Pluess, 2009; Boyce, 2016), but brain structure and function also appears as a viable mediator of such associations (Yap *et al.,* 2008; Whittle *et al.,* 2011; Schriber *et al.,* 2017; Deane *et al.,* 2019; Rifkin-Graboi *et al.,* 2019). However, very little research has been conducted on the brain structural factors that may underlie such susceptibility in young children. White matter microstructure, more specifically its diffusion properties, is related to 5-HTTLPR and BDNF Val66met genotypes (Tost *et al.,* 2013; Benedetti *et al.,* 2015; Tatham *et al.,* 2016), also linked to environmental susceptibility (e.g. Kim *et al.,* 2007). White matter microstructure is also reportedly related to developmental and psychiatric disorders (Barnea-Goraly *et al.,* 2005; Wolff *et al.,* 2012; Vulser *et al.,* 2018). Especially the white matter tracts that have a broad importance in connecting different areas of brain and the networks like default mode and salience network that have been indicated underlying variation in susceptibility (Greven *et al.,* 2019) may be viable mediators of susceptibility to environment. Thus, variation in white matter microstructure can be considered one potential neural phenotype underlying interindividual differences in susceptibility. Further, exposure to maternal pre- and post-natal depressive symptoms have been related to offspring white matter diffusion properties in both neonates and older children (Lebel *et al.,* 2016; Dean *et al.,* 2018; El Marroun *et al.,* 2018), suggesting an interplay between white matter microstructure, maternal perinatal depressive symptoms and child development.

The aim of the current study was to investigate whether the well-established association between maternal postpartum depressive symptoms and infant negative reactivity, a key trait reflecting higher risk for later psychopathology, is moderated by newborn white matter microstructure diffusion properties. We chose to focus broadly on mean fractional anisotropy (FA) in the whole brain as no previous studies with focus on white matter underlying the variation in susceptibility exist, and because in newborns, broad phenotypes including FA across the brain may have prominent implications for later development (Dubois *et al.,* 2014; Gilmore *et al.,* 2018; Girault *et al.,* 2019). We tested the association separately at 3 and 6 months to examine whether timing of exposure has relevance in determining child outcomes in the context of FA as a marker of susceptibility, and additionally, we conducted an analysis differentiating continuously elevated, low and discontinuously elevated (elevated only at either 3 or 6 months postpartum) maternal symptoms.

Furthermore, we explored the moderation effect more locally by focusing on the FA of the corpus callosum (CC), cingulum bundle (CB) and uncinate fasciculus (UF) tracts. CC is a key tract responsible for interhemispheric brain connectivity (Horn *et al.,* 2014; Roland *et al.,* 2017) and can be reliably delineated in the developing brain (Gilmore *et al.,* 2007; Jeurissen *et al.,* 2013; Qiu *et al.,* 2015). CC, CB and UF all also contribute to default mode and salience networks (Gordon *et al.,* 2011; Von der Heide *et al.,* 2013; Horn *et al.,* 2014; Bubb *et al.,* 2018), which are considered critical for the susceptibility to environment (Greven *et al.,* 2019). Furthermore, structural alterations in these tracts have been linked with prenatal and early life stress exposures, including maternal symptomatology (Jackowski *et al.,* 2008; Charil *et al.,* 2010; Rifkin-Graboi *et al.,* 2015; El Marroun *et al.,* 2018), emotion regulation (Vulser *et al.,* 2018) and a myriad of psychiatric disorders, including depression (Lacerda *et al.,* 2005; Arnone *et al.,* 2008; Walterfang *et al.,* 2008; Barnea-Goraly *et al.,* 2009; Bellani *et al.,* 2009; Koch *et al.,* 2014; Swartz *et al.,* 2014; Jenkins *et al.,* 2016; Tatham *et al.,* 2016), which in turn is predicted by heightened negative reactivity in childhood. Finally, white matter microstructure alterations, namely lower FA of CC and CB, have been reported in adults with treatment-resistant depression (De Diego-Adeliño *et al.,* 2014), further emphasizing the role of white matter microstructure in susceptibility to environmental influences. Although generally higher FA values reflect more advanced neural development (Gilmore *et al.,* 2018) and lower FA values are reported in clinical populations, also increased FA values may represent a risk, especially in pediatric populations (Barnea-Goraly *et al.,* 2005; Wolff *et al.,* 2012; Koch *et al.,* 2014; Bubb *et al.,* 2018). With no previous studies on the topic, we did not set an a priori hypothesis about whether lower or higher FA would reflect susceptibility to maternal symptoms of depression.

## Materials and methods

### Participants

The data are part of the FinnBrain Birth Cohort Study (Karlsson *et al.,* 2018), a prospective study starting from the prenatal period. A research nurse informed the families that underwent a first trimester ultrasound at gestational week 12 about the study, and of all the families informed, 66% enrolled in the study. A subset of families was invited to participate in the newborn brain magnetic resonance imaging (MRI) between 2 and 5 weeks of infant age. The families who participated in the newborn scan and whose parents also filled in the prenatal and 3- and 6-month postnatal questionnaires qualified for this study (*N* = 80 mother–infant dyads). The scanned infants were all born at gestational week 36 or later, weighed more than 2500 g, had Apgar scores >6 at 5 min after birth, did not have any diagnosed CNS anomaly or abnormal findings in the MRI scan, and had not undergone any invasive treatments after birth. Demographic characteristics of the sample are presented in Table 1. The sample of this study mainly resembled the main cohort (Karlsson *et al.,* 2018) but the mothers who did not respond to the 6-month questionnaire including the main outcome were less educated and had more depressive symptoms in mid-pregnancy than the mothers who provided the full data (see the Supplementary Material).

**Table 1:** The sample characteristics of mother–infant dyads (*N* = 80) in the study

Mother–infant dyads (*N* = 80)	Mean or No.	%	SD	range
Maternal age at childbirth	30.1		4.3	20–41
Duration of gestation	39.8		1.3	36–42
Depressive symptoms (EPDS)				
During pregnancy (averaged)	4.6		4.1	0–21
During postpartum (averaged)	4.6		3.9	0–15.5
3 months postpartum	4.4		4.0	0–19
of which score ≥ 10	6	9.4		
6 months postpartum	4.9		4.4	0–19
of which score ≥ 10	9	14.1		
Race/ethnicity, White/Caucasian	80	100		
Educational level				
High school/vocational	18	23		
Polytechnics	28	35		
University	34	42		
Maternal weight status (BMI) at gwk 14	24.3		4.2	17.5–38.4
Self-reported use of alcohol				
during first trimester	16	20		
during third trimester	5	6		
Self-reported smoking				
during first trimester	5	6		
during third trimester	2	3		
Parity				
Primiparous	49	61		
Multiparous	31	39		
Infant sex				
Male	40	50		
Female	40	50		
Infant age at scan from birth, days	24.3		8.0	11–54
Infant age at scan from conception (due date), days	306.0		7.2	293–323
Infant birth weight, grams	3540		461	2580–4700
APGAR score at 5 min after birth	9.13		0.65	6–10
Whole brain mean FA	0.25		0.02	0.22–0.30
Corpus callosum mean FA	0.32		0.02	0.27–0.39
Cingulum mean FA	0.43		0.05	0.19–0.50
Uncinate fasciculus mean FA	0.26		0.02	0.19–0.33
Infant negative reactivity at 6 months	2.98		0.80	1.48–4.89

### Ethical considerations

The study protocol was approved by the Ethics Committee of the Hospital District of Southwest Finland and was performed according to the Declaration of Helsinki. Parents gave informed written consent on behalf of themselves and their children.

## Measures

### Maternal depressive symptoms during pregnancy and at postpartum

Maternal depressive symptoms were measured using the Edinburgh Postnatal Depression Scale (EPDS; Cox *et al.,* 1987) that is also validated for use in the prenatal period (Bergink *et al.,* 2011). The questionnaire was filled in by mothers at gestational weeks 14, 24 and 34; at 3 months; and at 6 months (Cronbach’s *α* = 0.84–0.89). The EPDS consists of 10 items, each rated from 0 to 3, resulting in a maximum score of 30, higher scores indicating more depressive symptoms, and typically with 10 or greater indicating possible depression in community samples (e.g. Cox *et al.,* 1987; Eberhard-Gran *et al.,* 2001). Maternal depressive symptoms at 3 and 6 months postpartum were used as continuous variables in the main analysis. Further, an averaged sum of depressive symptoms throughout pregnancy was calculated to be used as a covariate in the analyses, as based on previous studies, prenatal distress could independently affect child brain (Scheinost *et al.,* 2017; Pulli *et al.,* 2018).

#### Continuity of maternal depressive symptoms

To examine the significance of exposure continuity, an additional analysis based on whether the mother had experienced depressive symptoms throughout the postnatal period or only at 3 or at 6 months was conducted. The classification was made based on the slightly lower EPDS score of 9 in comparison to the traditional threshold of 10 to maintain sufficient group sizes in the analyses and based on the literature showing that even subclinical symptoms are related to mother–infant interactions and infant outcomes (e.g. Campbell *et al.,* 1995; West and Newman, 2003; Moehler *et al.,* 2006; Giallo *et al.,* 2015). The mothers with this score or higher at both postpartum time points were considered to have continuously elevated symptoms. The classification resulted in *N* = 58 mothers with low symptoms across postpartum, *N* = 13 mothers with high symptoms across postpartum (both at 3 and 6 months), and *N* = 8 mothers with high symptoms only at 6 months. Only one mother reported high symptoms only at 3 but not at 6 months, so the classes including mothers with high symptoms only in either of the time points (discontinuously elevated symptoms) were collapsed into one class.

The descriptive statistics for maternal depressive symptoms are reported in Table 1.

### DTI-MRI data acquisition and processing

The scans were performed in the Medical Imaging Centre of Turku University Hospital in a family–friendly manner as previously described (Lehtola *et al.,* 2019). MRI scans were conducted on a Siemens Magnetom Verio 3 T scanner (Siemens Medical Solutions, Erlangen, Germany) using a 12-element Head Matrix coil. A 96-direction DTI protocol was divided into three parts (each part with either 31, 32 or 33 individual diffusion encoding directions using *b* = 1000 s/mm^2^ in addition to three *b* = 0 s/mm^2^ images, per each part, and a duration of approximately 6 min). In each part, the spread of diffusion encoding directions was evenly distributed across the 3D space. The sequences were acquired using Spin Echo-Echo Planar Imaging sequence at 2 mm^3^ isotropic resolution (FOV 208 mm; 64 slices; TR 8500 ms; TE 90 ms). Images were screened for incidental findings, and they did not affect white matter (Kumpulainen *et al.,* 2020).

### DTI data analysis

First, we visually identified b0 volumes with acceptable quality (no motion artifacts in visual inspection) and moved average of them to the front of the 4D series. We then created a brain mask from the average b0 volume with FSL’s FMRIB Software Library v 5.0.9 (Jenkinson *et al.,* 2012) Brain Extraction Tool (Smith, 2002). Second, the qualities of diffusion datasets were quantitatively evaluated using DTIprep software (Oguz *et al.,* 2014). Datasets were then formed from the three quality-controlled parts into single image with an in-house script. The combined data now included a variable number of diffusion encoding directions, and those containing less than 40 diffusion encoding directions were excluded from later analyses. Finally, directions in excess to 40 were removed, while always maximizing the angular resolution (Merisaari *et al.,* 2019), so that each participant now had exactly 40 diffusion encoding directions in their data. Finally, the 3D distribution of available diffusion encoding directions was assured to be even, as per planned coverage of the sequences and visual inspection of the distributions after preprocessing (Roalf *et al.,* 2016). Prior work points out that 40 directions provide a robust prerequisite for tensor estimation (Ni *et al.,* 2006; Giannelli *et al.,* 2010).

Next, we corrected the data for eddy currents using FSL (Andersson and Sotiropoulos, 2016). Correlation to minor residual motion (after dtiprep) was assessed in our prior work (Merisaari *et al.,* 2019) and they did not bias the estimates. Finally, we processed the 4D diffusion dataset with FSL’s dtifit, using the brain mask to limit the modeling to brain tissue only. All steps were followed by careful visual inspection of the data.

For further preprocessing, the tract-based spatial statistics (TBSS) pipeline of FSL (Smith *et al.,* 2007) was employed, limiting the analysis only to the skeletons of white matter tracts, estimated from individual images that are projected to a common skeleton space. The ‘tbss_2_reg -n’ option was used to create a study-specific template for spatial transformations. A modified version of the ‘tbss_3_postreg -S’ step was then run to incorporate registrations to the study-specific template and up-sample the data to 1 mm^3^ resolution as per TBSS defaults. An FA threshold of 0.15 in the ‘tbss_4_prestats’ module was used to create an FA skeleton (Merisaari *et al.,* 2019).

Finally, an automated, skeleton-based, ROI delineation was performed by masking the FA skeleton images with the JHU template (Oishi *et al.,* 2011). The JHU labels were warped to the study-specific infant mean FA space (see the Supplementary Material), and the JHU atlas was used to mask and estimate mean FA from the anatomical areas within the skeleton (all the tracts, including the CC and UF). The co-registration was visually inspected to assure accurate coverage of the labels for each of the FA skeletons. The whole brain mean FA was calculated as a mean of FA values in all white matter areas (inside the skeleton), and the mean FA’s of CC, (cingular part of) CB and UF were used in *post-hoc* analyses separately. Thus, the ROI values for the whole brain FA and the anatomical JHU labels/regions have been defined from the thresholded individual FA skeleton.

### Mother-reported infant negative reactivity

Maternal reports of the negative affectivity scale of the Infant Behavior Questionnaire Revised Short Form (IBQ-R; Putnam *et al.,* 2014) were used to measure negative reactivity at the infant age of 6 months. The negative affectivity scale (Cronbach’s *α* = 0.90) of the IBQ-R includes 25 items, where the parent assesses infant behaviors and expressions of distress, sadness, and fear during the past 1 or 2 weeks on a scale from 1 to 7, with higher scores on each scale indicating higher levels of negative reactivity.

### Statistical analyses

We evaluated the associations between relevant confounders, white matter FA in the whole brain, and mother-rated infant negative reactivity using Pearson correlations and pairwise *T*-tests, except for postnatal age and gestational age, which we tested using partial correlations controlling for each other. Because mother-reported infant negative reactivity was not found to deviate from normal distribution, we used linear regression models to analyze whether the interaction of newborn whole brain FA and maternal postnatal depressive symptoms at 3 and 6 months would predict infant negative reactivity at 6 months of age. The following covariates were included based on the previous literature on factors that may affect child brain and/or negative affect: infant sex (e.g. Else-Quest *et al.,* 2006), parity (Fish and Stifter, 1993), maternal alcohol/tobacco use during pregnancy (see a review in Pulli *et al.,* 2018) and maternal prenatal depressive symptoms (e.g. Dean *et al.,* 2018) and post-conceptional age, resulting in the following models:

Negative reactivity = infant sex + post-conceptional age + maternal parity + maternal alcohol/tobacco use during pregnancy + maternal EPDS_prenatal +_ whole brain FA + EPDS_postnatal_ + (EPDS_postnatal_ × whole brain FA), where EPDS_postnatal_ was either:

(a) EPDS at 3 months or (b) EPDS at 6 months.

We used logarithm-transformed and standardized maternal symptoms in the analyses. As a sensitivity analysis, we also conducted the model separately controlling for gestational age and postnatal age at scan instead of post-conceptional age to make sure that the age variable used did not affect the results (Rasmussen *et al.,* 2017). Further, we tested whether the results remain after controlling for several prenatal exposures (see the Supplementary Material). The main analyses were run using SPSS V25.0.

Finally, as a *post-hoc* analysis, we ran the analyses for the preselected regions of interest; CC, CB and UF. The *P*-values of the foci of interest interaction terms in all models (i.e. EPDS_3 months_ × FA and EPDS_6 months_ × FA in the main models and the following three *post-hoc* models) were corrected using the Benjamini–Hochberg method (Benjamini and Hochberg, 1995) using false discovery rate *P* < 0.05 threshold. Next, we probed interactions (Roisman *et al.,* 2012) for significant FA moderators using simple slopes using PROCESS Macro (Hayes, 2017) in SPSS. Thus, we tested whether maternal depressive symptoms and negative reactivity are associated at the low or high (± 1 SD) ends of the distribution of the FA mean (Aiken and West, 1991). Figures were made using the median split of whole brain FA and the package *ggplot2* in R (R Core Team, 2018). Finally, as white matter microstructure only interacted with 6-month maternal depression, we ran an ANCOVA model using continuity groups of maternal depressive symptoms to distinguish whether infant exposure to continuous high maternal distress *vs* high maternal distress at 6 months only was important in terms of infant negative reactivity as a function of white matter microstructure.

## Results

### Potential confounders, newborn whole brain fractional anisotropy, and infant negative reactivity

The correlations between the variables are shown in Table 2. Newborn whole brain FA correlated positively with post-conceptional age. Mother-rated negative reactivity was positively related to length of gestation and maternal parity (*T* = −2.17, *P* = 0.033, Cohen’s *d* = 0.49), with multiparous mothers rating their children higher in negative reactivity (*M* = 3.22, SD = 0.88) than primiparous mothers (*M* = 2.83, SD = 0.72). Whole brain FA or mother-rated negative reactivity was not significantly associated with depressive symptoms during pregnancy, child sex, or maternal alcohol or tobacco use during pregnancy (*P* > 0.05).

**Table 2:** Correlations between the whole brain mean FA, infant negative reactivity, maternal depressive symptoms at 3 and 6 months, and the covariates

	1	2	3	4	5	6	7
1 Whole brain FA							
2 Negative reactivity	0.10						
3 EPDS at 3 months	−0.04	0.38^**^					
4 EPDS at 6 months	−0.06	0.26^*^	0.69^**^				
5 Length of gestation	0.37^*^^a^	0.21	0.30^**^	0.26^*^			
6 Postnatal age at scan	0.39^**^^a^	−0.12	−0.26^*^	−0.18	−0.63^**^		
7 Age from conception	0.42^**^	0.11	0.05	0.05	0.49^**^	0.33^**^	
7 EPDS prenatal	0.08	0.13	0.64^**^	0.64^**^	0.15	-0.14	0.01

^*^ *P* < 0.05, FA = fractional anisotropy, EPDS = Edinburgh postnatal depression scale.

^**^ *P* < 0.01.

^a^A partial correlation adjusted for the other age variable (postnatal age for the length of gestation and length of gestation for postnatal age at scan).

### Maternal postnatal depressive symptoms, newborn whole brain fractional anisotropy, and infant negative reactivity

Maternal depressive symptoms at 3 or 6 months postpartum were not related to newborn whole brain FA values. Maternal depressive symptoms at 3 (*r* = 0.38, *P* = 0.001) and 6 months (*r* = 0.26, *P* = 0.019) were positively related to mother-rated infant negative reactivity. Newborn whole brain FA was not related to mother-rated infant negative reactivity.

### The interaction of maternal postpartum depressive symptoms and whole brain fractional anisotropy in predicting infant negative reactivity

**Fig. 1:**
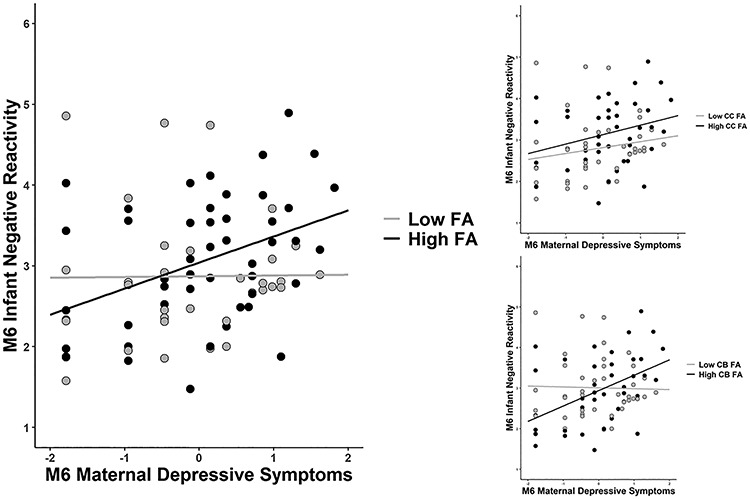
The association between maternal depressive symptoms at 6 months and infant negative reactivity at 6 months: moderation by low and high infant whole brain, CC and CB FA (groups based on median).

The interaction of whole brain FA and maternal postpartum depressive symptoms at 6 months predicted infant negative reactivity at 6 months, so that infants with higher FA exhibited more negative reactivity when exposed to increased maternal symptoms and less negative reactivity when exposed to low maternal symptoms (Table 3 and Figure 1). The interaction between maternal depressive symptoms at 3 months and newborn FA did not predict infant negative reactivity (see the Figure S2 in the Supplementary Material). Results remained significant regardless of the infant age variable included in the model, as well as when controlled for several prenatal exposures (see the Supplementary Material).

**Table 3:** The interaction of maternal postpartum depressive symptoms and newborn whole brain FA in predicting infant negative reactivity (*N* = 80)

	Model 1a 3-month EPDS		Model 1b 6-month EPDS			
	*B* (SE)	*P*	*B* (SE)	*P*	*P* adj.	∆*R*^2^
Step 1						
Infant sex	−0.04 (0.17)	0.795	−0.14 (0.18)	0.437		
Parity	0.36 (0.18)	0.045	0.38^*^(0.19)	0.047		
Alcohol/tobacco use	0.04 (0.20)	0.848	0.18 (0.21)	0.386		
Maternal EPDS (pregnancy)	−0.04 (0.03)	0.193	−0.01 (0.03)	0.702		
Age from conception	0.00 (0.01)	0.778	0.00 (0.1)	0.840		
Maternal EPDS (postpartum)	0.38 (0.12)	0.002	0.20 (0.12)	0.097		
Whole brain FA	6.04 (5.48)	0.274	5.96 (5.73)	0.302		
Whole brain FA × EPDS (postpartum)	4.13 (5.83)	0.48	18.20^**^ (6.27)	0.005	0.040	0.09^**^

^**^ *P* < 0.01, ^*^P < 0.05, all the beta coefficients and standard errors are unstandardized; the interaction terms in the main models and three sets of *post-hoc* models were corrected using the Benjamini–Hochberg method; ∆*R*^2^ refers to the significant interaction in Model 1b; the results are similar when postnatal age at scan and duration of gestation are controlled for separately.

### Post-hoc analyses: corpus callosum, cingulum bundle and uncinate fascicle

The interaction of maternal depressive symptoms at 6 months and CC FA (*B* = 10.55, *P* = 0.020) as well as CB FA (*B* = 5.21, *P* = 0.014) predicted infant negative reactivity in a similar manner to whole brain FA, while the interaction between the 3-month depressive symptoms and CC FA was not significant (*P* = 0.28–0.42). Maternal depressive symptoms in interaction with UF FA were not, however, found to predict infant negative reactivity (*P* > 0.05) (see detailed results in the Supplementary Material).

### Post-hoc analyses: probing the interaction

The simple slope analysis indicated that maternal depressive symptoms at 6 months were associated with higher infant negative reactivity when whole brain FA was high (= 0.27, *B* = 0.58 the 95% confidence interval = [0.23, 0.92], *P* = 0.001) or average (= 0.25, *B* = 0.2579 [0.03, 0.48], *P* = 0.026), but not when newborn FA was low (= 0.23, *B* = −0.06 [−0.34, 0.22], *P* = 0.68). Similar results were replicated for CC FA and CB FA as moderators (see the Supplementary Material).

### Post-hoc analyses: continuity of maternal symptoms

**Fig. 2:**
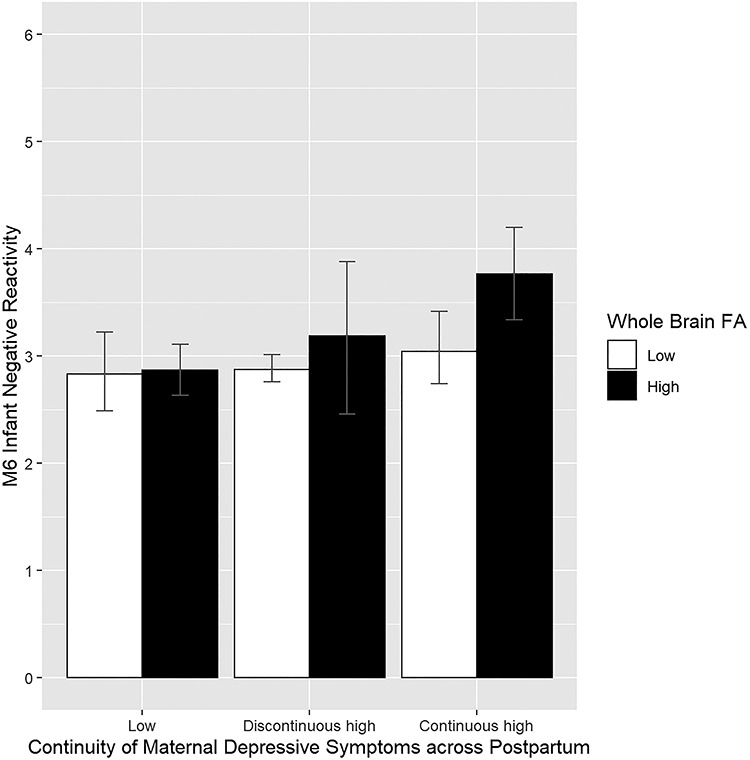
The continuity of maternal depressive symptoms across postpartum and infant negative reactivity within groups of low and high (based on median) whole brain FA. The error bars represent bootstrapped 95% confidence intervals of the group means.

We ran the analyses for whole-brain FA and the significant ROIs (CC and CB FA) using a grouping of maternal depressive symptoms based on the continuity of symptoms. All the analyses replicated a similar pattern of interactions as in the models where only the 6-month EPDS was a significant predictor together with infant FA values (see Table 4). Only the infants exposed to continuously high maternal symptoms (high maternal symptoms both at 3 and 6 months) differed from infants exposed to low symptoms (Figure 2). The infants exposed to symptoms only at 6 or at 3 months did not differ from those exposed to low maternal symptoms (when whole brain or CC FA were used as moderator) or either of the other groups (when CB was used as a moderator) in terms of their negative reactivity.

**Table 4:** The ANCOVA models testing interaction between white matter FA and maternal depressive symptom continuity across postpartum

	*B* (SE)	*P*	∆*ƞ*^2^
Continuity of symptoms		0.077	
Low *vs* continuous high	9.49 (4.17)	0.026	
Temporary *vs* continuous high	9.68 (5.41)	0.078	
Whole brain FA	41.39 (15.15)	0.046	
Whole brain FA × continuity		0.057	
**Low x FA *vs* High x FA**	**−39.56 (16.41)**	**0.019**	**0.08** ^*^
Temporary x FA *vs* High x FA	−40.29 (21.55)	0.066	0.05
Low x FA *vs* Temporary x FA	0.74 (16.60)	0.965	0.00
Continuity of symptoms		0.035	
Low *vs* continuous high	9.68 (3.67)	0.010	
Temporary *vs* continuous high	9.36 (5.02)	0.066	
CC FA	30.90 (10.59)	0.066	
CC FA x Continuity		0.24	
**Low x CC FA *vs* High x CC FA**	**−32.12 (11.48)**	**0.007**	**0.10** ^**^
Temporary x CC FA *vs* High x CC FA	−31.056	0.053	0.05
Low x CC FA *vs* Temporary x CC FA	−1.06 (12.41)	0.932	0.00
Continuity of symptoms		0.054	
Low *vs* continuous high	5.804 (2.08)	0.064	
Temporary *vs* continuous high	−0.463 (4.85)	0.924	
CB FA	10.23 (6.80)	0.137	
CB FA x Continuity		0.037	
**Low x CB FA *vs* High x CB FA**	**−14.53 (6.95)**	**0.040**	**0.06** ^**^
Temporary x CB FA *vs* High x CB FA	0.337 (11.18)	0.976	0.00
Low x CB FA *vs* Temporary x CB FA	−14.86 (8.95)	0.101	0.04

^*^ *P* < 0.05, ^**^P < 0.01. The significant interactions are bolded. All the beta coefficients and standard errors are unstandardized; all the models are controlled for parity, maternal depressive symptoms during pregnancy, child sex, post-conceptional age and tobacco/alcohol exposure during pregnancy.

## Discussion

The aim of the current study was to investigate whether newborn white matter microstructure measured as FA reflects infant sensitivity to maternal depressive symptoms with an outcome of infant negative reactivity at 6 months. Although maternal depressive symptoms were expectedly found to be related to negative reactivity overall, when examining the moderating effect of newborn whole brain FA, the association was found only among infants with average or high white matter FA as newborns, but not among the infants with low FA. Although the association was detected specifically between 6-month maternal symptoms and infant negative reactivity among infants with higher FA, further analyses suggested that infants with higher FA that were exposed to continuously elevated maternal depressive symptoms differed from those exposed to low or discontinuously high maternal depressive symptoms, and were at specific risk of showing heightened negative reactivity. This suggests that white matter FA may play a role in linking longer term exposure to maternal distress and infant reactivity, an association reported in previous studies (Giallo *et al.,* 2015; Prenoveau *et al.,* 2017). Similar findings were detected when using CC and CB FA, but not UF FA, as a moderator.

Better understanding of the interindividual differences in susceptibility to maternal mental health and subsequent offspring psychopathology has been stressed in the literature (Meaney, 2018). Moreover, conceptually similar results have been reported in adolescent populations linking limbic volumes or reduced frontal cortical thinning to susceptibility to the effects of parenting (Yap *et al.,* 2008; Whittle *et al.,* 2011; Deane *et al.,* 2019). Furthermore, a recent study reported larger newborn hippocampal volumes underlying infant susceptibility to maternal sensitivity in terms of later disorganized attachment style (Rifkin-Graboi *et al.,* 2019). The current study is thus among the first to report conceptually similar associations in early infancy when postnatal factors have only minimally shaped the brain, and the first to reports such associations with a focus on white matter metrics as a moderator. The study is of specific importance because white matter metrics typically show a mixed pattern of associations with several psychiatric and developmental outcomes. Our findings provide preliminary evidence for overall white matter FA underlying postnatal plasticity, proposing that high white matter FA at birth may moderate the sensitivity of the infant to the influence of continuously elevated levels of postpartum maternal distress. However, due to the small sample and the novelty of the findings, more research is needed to replicate the findings and make conclusions about the type of susceptibility.

Our finding that high newborn whole brain FA values were relevant for infant susceptibily in terms of negative reactivity was rather surprising, because lower FA values are typically related to psychopathology, including mood disorders (Barnea-Goraly *et al.,* 2005; Vulser *et al.,* 2018), which in turn are predicted by childhood negative reactivity. However, higher FA values have also been linked with behavioral problems in pediatric and adolescent populations (Wolff *et al.,* 2012; Koch *et al.,* 2014). Prenatal and early postnatal periods are periods of heightened neural sensitivity to environmental influences (Weiss and Wagner, 1998), such as stress generated by low parental mood and subsequent poorer quality of caregiving (Field, 2010). One possibility is that in some infants, higher FA values in the newborn period reflect more rapid neural development and premature myelination, which in turn makes them more susceptible to the current environment. This view is supported by studies linking neurotrophic factors to brain maturation and neuroplasticity in the postnatal period (Dyer *et al.,* 2016; Kowiański *et al.,* 2018), as well as a growing number of studies reporting that early adversity is associated with both accelerated brain maturation and a risk for later developmental problems (Callaghan and Tottenham, 2016; Posner *et al.,* 2016; Thijssen *et al.,* 2017; Spann *et al.,* 2019). Further, in line with our findings, one adult study has shown that lower FA values may reflect prolonged/treatment-resistant depression (less plasticity and response to environment) (De Diego-Adeliño *et al.,* 2014). Thus, FA metrics may reflect sensitivity to environment across different age groups.

We detected the association using CC and CB FA, but against our hypothesis, not using UF FA as moderators. Interestingly, CC and CB were among areas indicated to have decreased FA in adults with treatment-resistant depression, that is, less sensitivity to intervention (De Diego-Adeliño *et al.,* 2014). Moreover, white matter development is highly dynamic, and some studies have suggested that FA values in some areas may even decrease around birth (Gupta *et al.,* 2005; Bockhorst *et al.,* 2008). Thus, alternatively, higher FA values at a certain stage of infancy and in certain areas of the brain may also reflect atypical brain development and later risk for abnormal emotional development (Wolff *et al.,* 2012).

One further possibility not analyzed in the current study is that the relation between parental symptoms of depression and infant white matter FA is genetically determined (Carballedo *et al.,* 2012; Whalley *et al.,* 2013; Kochunov *et al.,* 2015). Additionally, epigenetic and other programming caused by parental symptoms of depression during pregnancy (Fatemi *et al.,* 2009; El Marroun *et al.,* 2018) and before conception could also affect newborn brain characteristics. As mentioned earlier, a growing number of studies have linked early life adversities to accelerated brain maturation (e.g. Gee *et al.,* 2013; Spann *et al.,* 2019). Thus, poorer parental mental health prior to and during pregnancy may program child brain development through epigenetic mechanisms toward more accelerated neurodevelopment reflected in white matter microstructure. The offspring with this neural phenotype may then also be programmed toward more susceptibility to the postnatal environment, and consequently, the behavior varies as a function of postnatal environment (parental distress) only within these offspring. In this study, the hypothesized interaction was found after controlling for several prenatal exposures. Given the lack of other similar studies, a more detailed understanding about the mechanisms underlying the findings is needed to make conclusions about the (epi)genetic basis of the findings.

In the present study, we had an adequate sample size for a newborn imaging study and wide coverage of validated questionnaire data. However, the findings of this study can only be considered preliminary for several reasons. First, we had relatively few mothers with low education and symptoms that could be considered clinically relevant, warranting ideally a replication in a sample with clinically depressed parents. Instead, our findings provide some insights into the neural mechanisms of normal variation in early negative reactivity, a precursor of later behavioral development. Second, postpartum depression is only a distant measure of environment and should optimally be considered together with measures of parenting or family well-being. Third, our analyses only focused on average FA values instead of larger brain networks or the other parameters of white matter microstructure, encouraging future studies to utilize simultaneous assessment of whole-brain connectivity. Fourth, major limitations are the maternal ratings of their own depressive symptoms as well as infant negative reactivity, leading to a possibility that mothers with higher symptoms are more prone to rate their infants’ distress (Richters and Pellegrini, 1989), although some studies also contradict the existence of this bias (Forman *et al.,* 2003).

We conclude that the association between maternal depressive symptoms, especially continuously elevated levels of symptoms, and infant negative reactivity, a key trait predicting later psychiatric disorders, was modulated by higher newborn whole brain FA. The findings, although still very preliminary, have important implications for studies of environmental sensitivity and differential susceptibility, suggesting that inter-individual variation in white matter microstructure at birth may underlie infant sensitivity to parental distress.

## Funding

This research work was supported by Alexander von Humboldt Foundation (S.N.), Emil Aaltonen Foundation (S.N. and J.J.T.), Hospital District of South-Western Finland State Research Grants for Clinical Research (J.J.T., H.M., N.S., L.K. and H.K.), Alfred Kordelin Foundation (J.J.T.), Turku University Foundation (J.J.T.), Sigrid Juselius Foundation (H.M.), Finnish Cultural Foundation (H.M.), Signe and Ane Gyllenberg Foundation (S.N., N.S., R.K., L.K. and HK), Yrjö Jahnsson Foundation (L.K.), the Brain and Behavior Research Foundation (YI Grant 19056; L.K.), Academy of Finland Research Council for Health (308176; L.K. and 253270, 264363 and 134950; H.K.), the Academy of Finland Research Council for Culture and Society (2608063; R.K.) and Jane and Aatos Erkko Foundation (H.K.).

## Declaration of interest

The authors declare no conflicts of interest.

## Supplementary Material

File008_nsaa081
