# Supplementary material for: Newborn white matter microstructure moderates the association between maternal postpartum depressive symptoms and infant negative reactivity
Source: Soc Cogn Affect Neurosci. 2020 Jun 24;15(6):649–60. doi: 10.1093/scan/nsaa081 (PMC7393309; doi:10.1093/scan/nsaa081)
Supplement: File008_nsaa081 [file file008_nsaa081.docx]

**Supplementary Material: Additional Analyses**

**1. Attrition analyses**

The mothers who provided all the data had fewer depressive symptoms in mid-pregnancy (*T* = 2.53, *p* = .015), and were more highly educated (*χ² [1]* = 6.10, *p* = .047, *ƞ²* = .18) than non-responder mothers. Responding mothers were not different in terms of age, their infant’s sex, duration of gestation or parity, newborn CC FA values or depressive symptoms in other time points (*p* > .05). From all the families where a father was involved in the study, fathers who provided all data had infants with a longer duration of gestation (*T* = –2.01, *p* = .048) and higher overall CC FA values (*T* = –1.79–2.35, *p* = .021–.080). The responding fathers were not different in terms of age, education, depressive symptoms or their infant’s sex in comparison to non-responding fathers (*p* > .05). No information was available from the subject families that declined to participate in the beginning, but the characteristics of the sample and the whole cohort relatively well correspond to the demographics of the families in the area (Karlsson et al., 2018).

Karlsson, L., Tolvanen, M., Scheinin, N. M., Uusitupa, H. M., Korja, R., Ekholm, E., … Karlsson, H. (2018). Cohort Profile: The FinnBrain Birth Cohort Study (FinnBrain). International Journal of Epidemiology, 47(1), 15-16j. <https://doi.org/10.1093/ije/dyx173>

**2. The study-specific FA template**

**
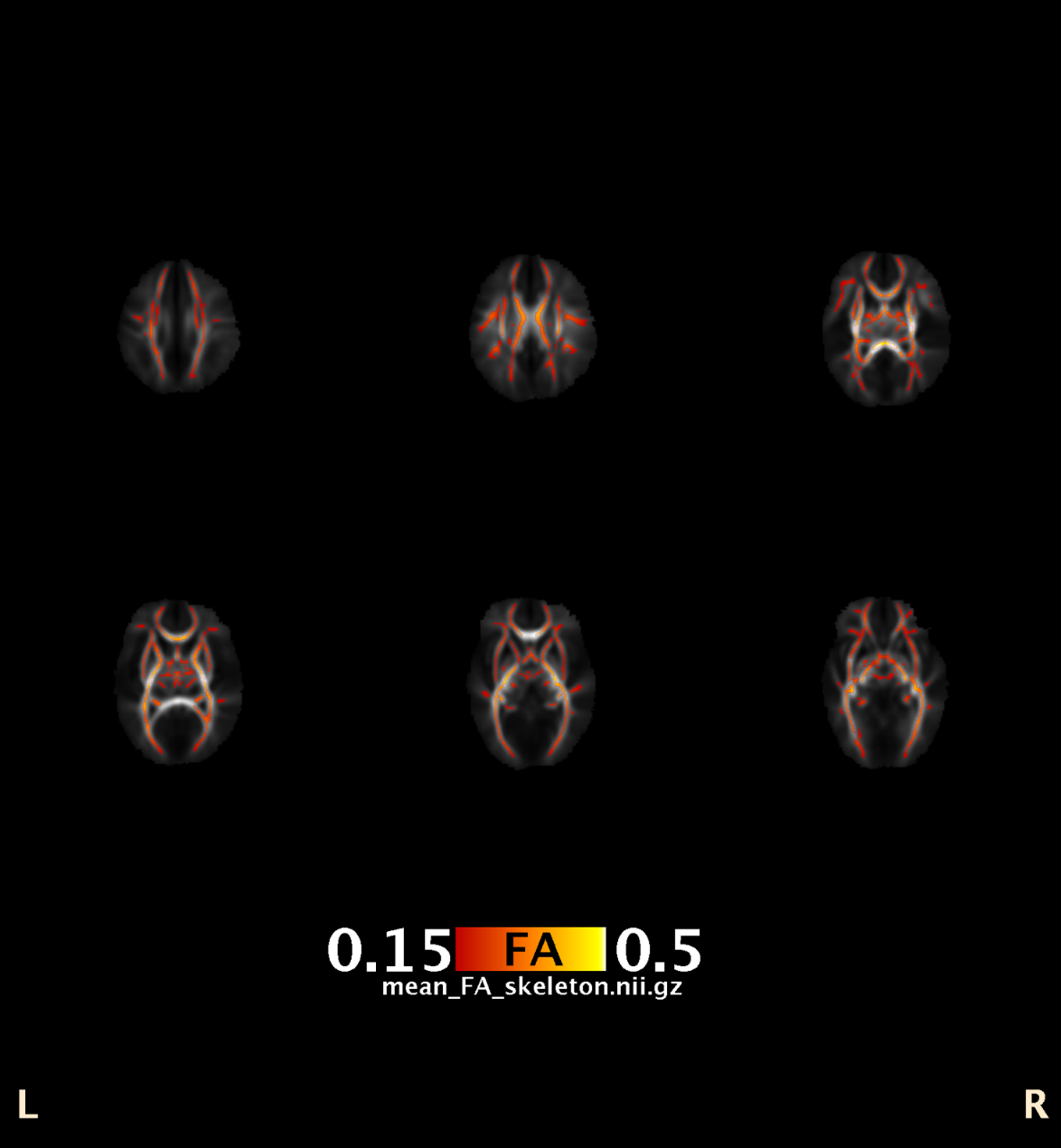
**

**Figure S1.** The study-specific template (mean FA image) and the mean FA skeleton as an overlay.

**2. Supplementary Figure: Maternal Symptoms at 3 Months, Infant Negative Reactivity and Whole Brain FA**

The association between maternal symptoms at 3 months and infant negative reactivity moderated by high and low corpus whole brain FA is displayed in **Figure S2**.

**Figure S2.** The Association between Maternal Symptoms at 3 Months Infant Negative Reactivity at 6 Months Moderated by High and Low Whole Brain FA Groups (Split Based on Median)

**3. The Consideration of Other Prenatal Risk Factors and Important Covariates: Maternal Anxiety, Obstetric Complications and APGAR scores**

All the maternal and paternal models were tested also controlling for prenatal anxiety (measured using Symptom Checklist -90 anxiety subscale (Derogatis, Lipman, & Covi, 1973) or Pregnancy-Related Anxiety Questionnaire Revised 2 (Huizink et al., 2015) and obstetric complications (0 = no obstetric complications, 1 = any obstetric complication during pregnancy). Further, the models were sensitivity tested controlling for APGAR scores at 5 minutes. None of these variables affected the model when included as covariates, suggesting that the observed associations were found regardless of the prenatal risk factors and newborn status at birth.

Derogatis, L. R., Lipman, R. S., & Covi, L. (1973). SCL-90: an outpatient psychiatric rating scale - preliminary report. *Psychopharmacology Bulletin*, *9*(1), 13–28. Retrieved from http://www.ncbi.nlm.nih.gov/pubmed/4682398

Huizink, A. C., Delforterie, M. J., Scheinin, N. M., Tolvanen, M., Karlsson, L., & Karlsson, H. (2015). Adaption of pregnancy anxiety questionnaire–revised for all pregnant women regardless of parity: PRAQ-R2. *Archives of Women’s Mental Health*, 125–132. https://doi.org/10.1007/s00737-015-0531-2

**4. Post-Hoc Analyses: The Interaction between Corpus Callosum FA, Cingulum Bundle FA and Uncinate Fasciculus FA and Maternal Depressive Symptoms in Predicting Infant Negative Reactivity**

The similar results as with whole brain FA were replicated when using CC and CB FA as a predictor, but not when using UF FA as a predictor (see Tables S1–S3). The simple slope analysis indicated that maternal depressive symptoms at 6 months were associated with higher infant negative reactivity when CC FA was high (=.3416, B = 0.4902 [0.15, 0.83], p = .0049) or average (=.3175, B = 0.2351 [0.01, 0.47], p = .0453) but not when newborn CC FA was low (=.2933, B = -0.0200 [–.31, .27], p = .8907). Similarly, the association was detected when CB FA was high (=.4770, B = 0.5035 [0.19, 0.82], p = .0020), but not when CB FA was average (= 0.4254, B = 0.2070 [-0.01, 0.43], p = .0663) or low (=0.3739, B = -0.0895 [-0.41, 0.23] p = .5737). The association between 6-month EPDS and infant negative reactivity within groups of low and high CC and CB FA (of each tract, based on median) are displayed in main manuscript Figure 1.

|  | Model 1a  3-month EPDS | | Model 1b  6-month EPDS | | | |
| --- | --- | --- | --- | --- | --- | --- |
|  | *B* (SE) | *p* | *B* (SE) | *p* | p adj. | ∆*R*² |
| Step 1 |  |  |  |  |  |  |
| Infant sex | -0.04 (0.17) | .835 | -0.14 (.17) | .428 |  |  |
| Parity | 0.33 (0.18) | .069 | 0.33 (0.18) | .089 |  |  |
| Alcohol/tobacco use | 0.05 (0.20) | .817 | 0.24 (0.20) | .236 |  |  |
| Maternal EPDS (pregnancy) | -0.04 (0.03) | .194 | -0.02 (0.03) | .405 |  |  |
| Age from conception | 0.01 (0.01) | .454 | 0.01 (0.1) | .361 |  |  |
| Maternal EPDS (postpartum) | -0.66 (1.30) | .614 | -3.12 (1.39) | .028 |  |  |
| CC FA | 2.06 (3.90) | .599 | 1.59 (3.90) | .685 |  |  |
| CC FA x EPDS (postpartum) | 3.30 (4.11) | .424 | 10.55* (4.43) | .020 | 0.05 | .06* |
| ** *p* < .01, * *p* < .05, all the beta coefficients and standard errors are unstandardized; the two interaction terms that are in the focus of interest were corrected using the Benjamini-Hochberg method; ∆R² refers to the significant interaction in Model 1b; the results are similar when postnatal age at scan and duration of gestation are controlled for separately. | | | | | | |

**Table S1.** The Interaction between CC FA and Maternal Depressive Symptoms in Predicting Infant Negative Reactivity at 6 Months

**Table S2**. The Interaction of Cingulum Bundle FA and Maternal Depressive Symptoms in Predicting Infant Negative Reactivity at 6 Months

|  | Model 1a  3-month EPDS | | Model 1b  6-month EPDS | | | |
| --- | --- | --- | --- | --- | --- | --- |
|  | *B* (SE) | *p* | *B* (SE) | *p* | p adj. | ∆*R*² |
| Step 1 |  |  |  |  |  |  |
| Infant sex | -0.08 (0.17) | 0.66 | -0.14 (0.17) | 0.39 |  |  |
| Parity | 0.32 (0.17) | 0.07 | 0.38 (0.18) | 0.03 |  |  |
| Alcohol/tobacco use | 0.04 (0.20) | 0.84 | 0.22 (0.20) | 0.26 |  |  |
| Maternal EPDS (pregnancy) | -0.03 (0.03) | 0.27 | -0.02 (0.03) | 0.39 |  |  |
| Age from conception | 0.01 (0.01) | 0.25 | 0.01 (0.01) | 0.36 |  |  |
| Maternal EPDS (postpartum) | -0.43 (0.72) | 0.55 | -2.24 (0.93) | 0.02 |  |  |
| CB FA | -1.55 (1.83) | 0.40 | -0.23 (2.05) | 0.91 |  |  |
| CB FA x EPDS (postpartum) | 1.89 (1.69 | 0.27 | 5.75 (2.17) | 0.01 | 0.04 | 0.08** |
| ** *p* < .01, * *p* < .05, all the beta coefficients and standard errors are unstandardized; ; the interaction terms in the main models and three sets of post-hoc models were corrected using the Benjamini-Hochberg method ; ∆R² refers to the significant interaction in Model 1b; the results are similar when postnatal age at scan and duration of gestation are controlled for separately. | | | | | | |

**Table S3.** The Interaction of Uncinate Fasciculus FA and Maternal Depressive Symptoms in Predicting Infant Negative Reactivity at 6 Months

|  | Model 1a  3-month EPDS | | Model 1b  6-month EPDS | | | |
| --- | --- | --- | --- | --- | --- | --- |
|  | *B* (SE) | *p* | *B* (SE) | *p* |  |  |
| Step 1 |  |  |  |  |  |  |
| Infant sex | -0.04 (0.17) | .796 | -0.13 (0.17) | .472 |  |  |
| Parity | 0.35 (0.18) | .051 | 0.39 (0.19) | .038 |  |  |
| Alcohol/tobacco use | 0.05 (0.20) | .809 | 0.17 (0.21) | .410 |  |  |
| Maternal EPDS (pregnancy) | -0.03 (0.03) | .282 | -0.01 (0.03) | .869 |  |  |
| Age from conception | 0.00 (0.01) | .868 | 0.00 (0.1) | .890 |  |  |
| Maternal EPDS (postpartum) | -0.21 (1.00) | .837 | 6.88 (0.20) | .146 |  |  |
| UF FA | 5.89 (4.44) | .189 | -1.11 (1.05) | .295 |  |  |
| UF FA x EPDS (postpartum) | 2.23 (3.92) | .571 | 5.11 (4.20) | .227 |  |  |
| All the beta coefficients and standard errors are unstandardized; ; the interaction terms in the main models and three sets of post-hoc models were corrected using the Benjamini-Hochberg method; ∆R² refers to the significant interaction in Model 1b; the results are similar when postnatal age at scan and duration of gestation are controlled for separately. | | | | | | |
